# Editorial: Less and Non-invasive Hemodynamic Monitoring Techniques

**DOI:** 10.3389/fmed.2018.00258

**Published:** 2018-09-19

**Authors:** Bernd Saugel, Samir G. Sakka

**Affiliations:** ^1^Department of Anesthesiology, Center of Anesthesiology and Intensive Care Medicine, University Medical Center Hamburg-Eppendorf, Hamburg, Germany; ^2^Department of Anesthesiology and Operative Intensive Care Medicine, University of Witten/Herdecke, Cologne Merheim Medical Center, Cologne, Germany

**Keywords:** cardiovascular dynamics, cardiac output, blood pressure, intensive care medicine, anesthesiology, goal-directed therapy

The measurement or estimation of hemodynamic variables reflecting blood pressure, blood flow, cardiac contractility, cardiac preload, and cardiac afterload plays a pivotal role in the monitoring, diagnostic workup, and treatment of critically ill patients treated in the intensive care unit or in patients having major surgery.

Besides pulmonary artery catheterization that is established as a classical method to observe, measure, and derive a variety of variables reflecting cardiovascular and oxygen dynamics (1, 2) a variety of “modern” less- and non-invasive hemodynamic monitoring methods became available during the last decades (3, 4). The physical measurement principles, clinical applications, and limitations of these technologies are discussed in a series of articles that are part of the research topic “Less- and Non-invasive Hemodynamic Monitoring Techniques.”

As a very basic hemodynamic variable and as a main determinant of the organs' perfusion pressure, arterial blood pressure is part of routine monitoring in intensive care medicine and anesthesiology. In a narrative review article, methods for non-invasive intermittent and continuous blood pressure monitoring are summarized (Meidert and Saugel). The authors recommend monitoring of blood pressure with intermittent oscillometry in hemodynamically stable, low-risk patients. In surgical patients at risk for hemodynamic instability, continuous non-invasive blood pressure monitoring with innovative techniques might become an option in the near future. In critically ill and high-risk surgical patients, continuous invasive blood pressure monitoring with an arterial catheter will be the method of choice in the foreseeable future. Based on these general recommendations, an article by Stenglova and Benes describes the evidence for the use of continuous non-invasive blood pressure monitoring during surgery and its potential to improve postoperative outcome by an early recognition (or even prediction) of hypotension.

In addition to blood pressure, the analysis of the arterial blood pressure waveform (pulse wave analysis) enables stroke volume, cardiac output, and dynamic cardiac preload parameters to be assessed using invasive (arterial catheter) or non-invasive (finger cuff) methods. Several articles discuss pulse wave analysis and its use in clinical practice.

Grensemann comprehensively explains the basic measurement principle of commercially available invasive monitoring systems using pulse wave analysis to estimate cardiac output. He emphasizes that pulse wave analysis is limited in patients with altered vascular tone and that uncalibrated systems should be used to follow cardiac output changes (trend monitoring) rather than to guide therapy based on absolute values of cardiac out. A review article by Yamada et al. describes how minimally invasive and non-invasive hemodynmaic monitoring techniques can be used to guide perioperative hemodynamic therapy and eventually improve postoperative outcome in surgical patients. The authors conclude that “monitoring equipment that can provide precise hemodynamic information without the complications and complexity of invasive techniques can facilitate individualized hemodynamic management and lead to improved outcomes.”

In addition to this narrative review, a perspective article by Saugel and Reuter focuses on the use of invasive uncalibrated pulse wave analysis for perioperative hemodynamic management (often referred to as “perioperative goal-directed therapy”). The article briefly summarizes the evidence and concludes that perioperative goal-directed therapy based on pulse wave analysis-derived blood flow and dynamic cardiac preload variables can improve patient outcome in high-risk patients. This conclusion is in line with the results of recent meta-analyses showing that perioperative goal-directed therapy seems to reduce postoperative morbidity (5, 6). However, further well-designed and adequately powered studies are needed to answer open questions about optimal target variables and values and about how to implement these perioperative treatment strategies in clinical routine. Another perspective article written by Nicklas and Saugel discusses current evidence and open research questions related to completely non-invasive hemodynamic monitoring methods for perioperative hemodynamic management. Nguyen and Squara provide an in-depth review of non-invasive methods to estimate cardiac output besides pulse wave analysis. They explain the basic measurement principles and validation data of bioimpedance/bioreactance, partial carbon dioxide rebreathing, pulse wave transit time, ultrasonic methods, and inductance thoracocardiography. In particular, the authors focus on the feasibility of these methods in the intensive care unit setting and emphasize that hemodynamic monitoring of critically ill patients requires good measurement performance in terms of accuracy, precision, and step-response change. They conclude that “further developments are needed to provide clinicians with sufficiently accurate devices for routine use.”

Optimization of oxygen delivery to the end-organs is the ultimate goal of therapeutic interventions aiming at an optimization of global cardiovascular dynamics. Accordingly, Molnar and Nemeth emphasize that—in addition to global blood flow variables such as stroke volume or cardiac output– markers of tissue oxygenation need to be considered during resuscitation of patients with circulatory shock. They explain the (patho)physiology of oxygen delivery, consumption, and extraction and discuss the value of central venous oxygen saturation to individually tailor therapeutic interventions to the individual patient's needs. The authors advocate for multimodal and individualized hemodynamic treatment strategies which should integrate various physiological variables (e.g., central venous oxygen saturation, lactate, venous-to-arterial carbon dioxide gap).

In summary, this series of articles reflects that a variety of innovative less- and non-invasive methods for advanced hemodynamic monitoring in intensive care and perioperative medicine are currently available. Future research needs to confirm that goal-directed optimization of global hemodynamics based on advanced less- and non-invasive hemodynamic monitoring can eventually improve oxygen delivery and have beneficial impact on patient outcome.

## Author contributions

BS and SGS drafted the manuscript and approved the final version of the manuscript to be published.

### Conflict of interest statement

BS and SGS collaborate with Pulsion Medical Systems SE (Feldkirchen, Germany) as members of the medical advisory board and received honoraria for giving lectures and refunds of travel expenses from Pulsion Medical Systems SE. BS received research support from Edwards Lifesciences (Irvine, CA, USA). BS received institutional research grants, unrestricted research grants, and refunds of travel expenses from Tensys Medical Inc. (San Diego, CA, USA). BS received honoraria for giving lectures from CNSystems Medizintechnik AG (Graz, Austria). BS received refunds of travel expenses from CNSystems Medizintechnik AG (Graz, Austria).

## References

[1] Gidwani UK Mohanty B Chatterjee K. The pulmonary artery catheter: a critical reappraisal. Cardiol Clin. (2013). 31:545–65. 10.1016/j.ccl.2013.07.008 24188220

[2] Cholley BP. Benefits, risks and alternatives of pulmonary artery catheterization. Curr Opin Anaesthesiol. (1998) 11:645–50. 17013285 10.1097/00001503-199811000-00010

[3] Teboul JL Saugel B Cecconi M De Backer D Hofer CK Monnet X. Less invasive hemodynamic monitoring in critically ill patients. Intensive Care Med. (2016) 42:1350–9. 10.1007/s00134-016-4375-7 27155605

[4] Saugel B Cecconi M Wagner JY Reuter DA. Noninvasive continuous cardiac output monitoring in perioperative and intensive care medicine. Br J Anaesth. (2015) 114:562–75. 10.1093/bja/aeu447 25596280

[5] Chong MA Wang Y Berbenetz NM McConachie I. Does goal-directed haemodynamic and fluid therapy improve peri-operative outcomes? a systematic review and meta-analysis. Eur J Anaesthesiol. (2018) 35:469–83. 10.1097/EJA.0000000000000778 29369117

[6] Pearse RM Harrison DA MacDonald N Gillies MA Blunt M Ackland G. (2014) Effect of a perioperative, cardiac output-guided hemodynamic therapy algorithm on outcomes following major gastrointestinal surgery: a randomized clinical trial and systematic review. JAMA 311:2181–90. 10.1001/jama.2014.5305 24842135

